# Supplementary figures and images for: Lampenflora in a Show Cave in the Great Basin Is Distinct from Communities on Naturally Lit Rock Surfaces in Nearby Wild Caves
Source: Microorganisms. 2021 May 31;9(6):1188. doi: 10.3390/microorganisms9061188 (PMC8227912; doi:10.3390/microorganisms9061188)

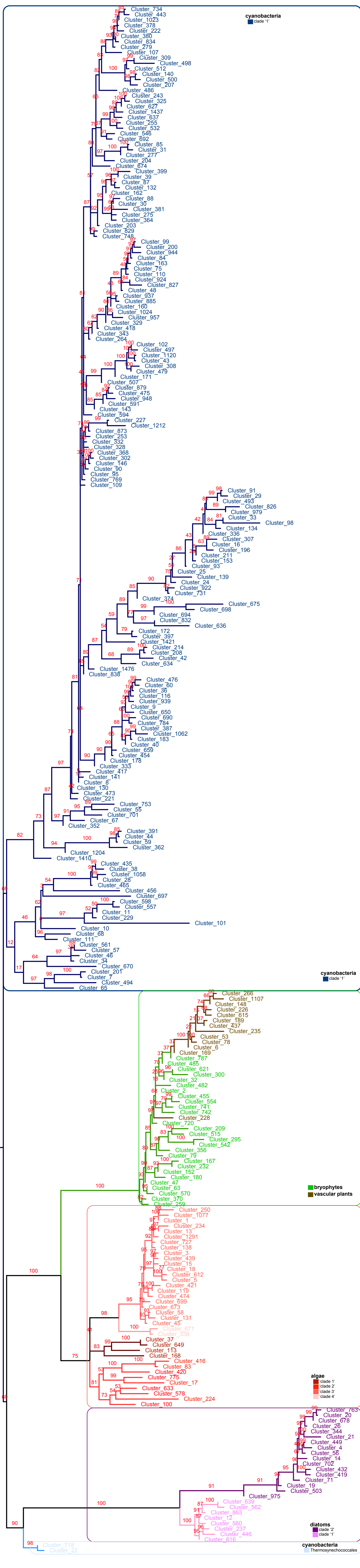

Supplement: Supplementary file 1 [file microorganisms-09-01188-s001.zip › supplementary_files/Fig_S1_23S_phylogenyR1.pdf]
