# Supplementary material for: Lampenflora in a Show Cave in the Great Basin Is Distinct from Communities on Naturally Lit Rock Surfaces in Nearby Wild Caves
Source: Microorganisms. 2021 May 31;9(6):1188. doi: 10.3390/microorganisms9061188 (PMC8227912; doi:10.3390/microorganisms9061188)
Supplement: Supplementary file 1 [file microorganisms-09-01188-s001.zip › supplementary_files/supplementary_file_S1_23S_photoautotrophs_FROGS_R1.zip!/Algae/htmls/01-prepro-vsearch.html]

FROGS Pre-process


javascript is needed to display data.  
If you try to view this data on galaxy please contact your administrator to authorise javascript or download the file to view.

## Preprocess summary

## Details on merged sequences

With selection:
 Display amplicon lengths 
 Display preprocessed amplicon lengths

## Details on artificial combined sequences

#### Amplicons lengths

×

Close
