# Supplementary material for: Lampenflora in a Show Cave in the Great Basin Is Distinct from Communities on Naturally Lit Rock Surfaces in Nearby Wild Caves
Source: Microorganisms. 2021 May 31;9(6):1188. doi: 10.3390/microorganisms9061188 (PMC8227912; doi:10.3390/microorganisms9061188)
Supplement: Supplementary file 1 [file microorganisms-09-01188-s001.zip › supplementary_files/supplementary_file_S1_23S_photoautotrophs_FROGS_R1.zip!/Algae/htmls/08-clustersStat.html]

FROGS Cluster stat


javascript is needed to display data.  
If you try to view this data on galaxy please contact your administrator to authorise javascript or download the file to view.

- Clusters distribution
- Sequences distribution
- Samples distribution

## Sequences count

## Hierarchical clustering
